# Efficacy of De‐Epithelialized vs. Subepithelial Grafts in Periodontal Plastic Surgery for Gingival Recession: A Systematic Review and Meta‐Analysis of Root Coverage Techniques

**DOI:** 10.1002/cre2.70499

**Published:** 2026-11-09

**Authors:** Lucrezia Parma Benfenati, Saeed Abdelaziz ElRefaei, Muhammad H. A. Saleh, Hom‐Lay Wang

**Affiliations:** ^1^ Department of Periodontics and Oral Medicine University of Michigan School of Dentistry Ann Arbor Michigan USA

**Keywords:** connective tissue graft, gingival recession, meta‐analysis, patient‐reported outcome measures, periodontal attachment loss

## Abstract

**Objectives:**

To compare the efficacy of de‐epithelialized gingival graft (DGG) versus subepithelial connective tissue grafts (SCTG) for the treatment of RT1 gingival recessions.

**Materials and Methods:**

Five databases were searched with no restrictions for randomized clinical trials (RCTs) directly comparing DGG and SCTG for treating RT1 gingival recessions. Primary outcomes were mean recession coverage (mRC) and complete root coverage (CRC%). Secondary outcomes included keratinized tissue width (KTW) gain, clinical attachment level (CAL) gain, probing depth (PD) change, Root Coverage Esthetic Score (RES), and patient‐reported pain/discomfort (PROs). Random‐effects models (REML) with Hartung–Knapp adjustment were used to calculate mean differences (MDs) with 95% confidence intervals (CIs).

**Results:**

Six RCTs (*n* = 221 patients) were included. No significant difference was observed in mRC (MD = −0.51 mm; 95% CI: −2.06 to 1.03; *p* = 0.368; *I* ^2^ = 95.9%). CRC significantly favored DGG (MD = 4.45%; 95% CI: 1.21–7.69; *p* = 0.027; *I* ^2^ = 0%). Small differences in KTW gain and PD reduction favored DGG, although heterogeneity was substantial for PD change. RES and PROs showed no consistent differences.

**Conclusions:**

Both DGG and SCTG are effective for correction of RT1 recessions. DGG showed modest advantages in CRC and PD reduction, while other outcomes were comparable.

**Clinical Significance:**

Both de‐epithelialized gingival grafts and subepithelial connective tissue grafts provide predictable root coverage for RT1 gingival recessions. De‐epithelialized grafts may offer a modest advantage in achieving complete root coverage without compromising esthetics or patient comfort, supporting their use as a viable alternative in periodontal plastic surgery.

## 1 Introduction

Surgical root coverage for gingival recession is the treatment of choice in cases of esthetic concern, root caries prevention, or hypersensitivity reduction, and to improve gingival phenotype (Tavelli et al. 2019).

The first palatal harvesting technique was proposed by Nabers in 1966 (Nabers 1966), to minimize the invasiveness of the graft, followed by several modifications such as Edel's “trapdoor” (Edel 1974), the Langer and Langer technique (Langer and Langer 1985), the J Bruno mixed graft (Bruno 1994), and the single‐incision approach. The last technique minimizes morbidity and ensures a consistent graft thickness, harvesting a “deeper” connective tissue graft (CTG) including the periosteum layer (Lorenzana and Allen 2000; Hürzeler and Weng 1999), and making single‐incision subepithelial connective tissue grafts (SCTGs) the gold standard for treating RT1 gingival recessions (Miller Class I/II) (Chambrone et al. 2008; Parma‐Benfenati et al. 2025).

Although secondary healing of the palatal donor site following epithelialized CTG harvesting may increase postoperative morbidity (Del Pizzo et al. 2002), a later study suggested that postoperative pain is more strongly associated with graft thickness than with the type of healing (Zuhr et al. 2014).

The de‐epithelialized connective tissue graft (DGG), introduced by Zucchelli et al. (2010), allows harvesting regardless of palatal thickness and yields denser, more stable connective tissue with minimal fatty or glandular content (Zucchelli et al. 2010; Bertl et al. 2015). As epithelial characteristics depend on the underlying connective tissue, DGG composition may enhance keratinization and reduce postoperative shrinkage (Bertl et al. 2015). Although free gingival grafts healing by secondary intention can increase morbidity (Farnoush 1978), later studies showed that pain is mainly related to graft thickness rather than healing type (Zucchelli et al. 2010).

According to the recent consensus on esthetic and patient‐reported outcomes in periodontology (Tonetti et al. 2025) studies on root coverage should evaluate both clinician‐reported outcomes (ClinROs) such as %CRC, %mRC, CAL gain, KTW change, gingival thickness (GT) change, and esthetic scores using validated tools like RES (Cairo et al. 2009); as well as patient‐reported pain/discomfort (PROs) and patient‐reported experiences (PREs) (Needleman et al. 2023).

Therefore, this systematic review aims to compare the efficacy of DGG versus SCTG in bilaminar root coverage techniques for treating RT1 gingival recessions.

## 2 Materials and Methods

### 2.1 Registration and Reporting Format

This systematic review was developed following a focused research question and designed and reported in accordance with the Preferred Reporting Items for Systematic Reviews and Meta‐Analyses (PRISMA) guidelines (Liberati et al. 2009). The protocol was prospectively registered with the International Prospective Register of Systematic Reviews (PROSPERO) under n. 1108275.

***Focused Question:** What is the comparative efficacy of de‐epithelialized gingival grafts (DGG) versus SCTGs when used in root coverage procedures for the treatment of Cairo RT1 (*Cairo et al. 2009) *(Miller Class I/II) (*Miller 1985a) *gingival recessions, regardless of the recipient‐site surgical technique employed?*

Based on a previous Cochrane systematic review assessing the efficacy of root coverage procedures (Chambrone et al. 2018), the following PICO framework was designed to address this review's focused question:

#### 2.1.1 Pico Framework

**P (Population):** Adult patients with single or multiple RT1 (Miller Class I or II) gingival recession defects.

**I (Intervention):** Root coverage procedures using de‐epithelialized gingival grafts (DGG), with any mucogingival surgical technique.

**C (Comparison):** Root coverage procedures using SCTGs with any mucogingival surgical technique.

**O (Outcomes):**

***Primary outcomes:***

Clinros: Mrc (Mm and Percentage) and Crc (Percentage).

Patient‐reported pain/discomfort (PROs): Pain scores using Visual Analog Scale (VAS) or Numeric Rating Scale (NRS), changes in dentin hypersensitivity, healing time, OHRQol, and overall satisfaction.

***Secondary outcomes:***

ClinROs: CAL gain, PD change, KTW change, GT change, and esthetic outcomes (e.g., RES).

### 2.2 Eligibility Criteria

Randomized clinical trials (RCTs); human subjects with maxillary or mandibular RT1/Miller Class I or II gingival recession; root coverage procedures comparing DGG with SCTG; reporting at least one relevant clinical outcome.

#### 2.2.1 Types of Interventions Included

- LPF: Laterally positioned flap
- CAF: Coronally advanced flap
- TUN: Tunnel flap technique
- SCTG + LPF/CAF/TUN
- Technical variations: with/without vertical releasing incisions

#### 2.2.2 Exclusion Criteria

Non‐randomized controlled studies, or retrospective studies; studies combining CTG with adjunctive biologic agents (e.g., EMD, PRP); studies lacking a clear description of graft harvesting technique; mucogingival surgeries without bilaminar techniques; recession RT2 and RT3 (Miller Classes III and IV) or with interproximal bone loss; studies with defects unrelated to gingival recession (e.g., non‐cervical carious lesions).

### 2.3 Search Methods for Included Studies

A comprehensive literature search was conducted across electronic databases (Scopus, MEDLINE/PubMed, Embase, Web of Science, and Gray literature); reference lists of the selected studies to identify any additional eligible articles; experts will also be consulted to improve search findings. A detailed search strategy using Boolean operators, MeSH terms, and free‐text terms specific to root coverage surgical techniques and CTG, comparing the efficacy of two harvested grafts. Example terms include “gingival recession,” “mucogingival surgery,” “coronally advanced flap,” “de‐epithelialized gingival graft,” “sub‐epithelial connective tissue graft,” combined with operators such as AND/OR.

Additionally, an electronic search was performed by screening articles published in relevant scientific journals with no time restrictions (i.e., *Journal of Clinical Periodontology*, *Journal of Periodontology*, *BMC, Journal of Periodontal Research*, and *Clinical Oral Investigations*), as well as any systematic reviews on this topic (Tavelli et al. 2019).

The complete search strategy for each database and the PRISMA flowchart are provided in Appendix A. No restrictions were placed on publication date and language.

### 2.4 Selection Process, Data Collection, and Management

All identified records were imported into the Rayyan online AI platform (https://new.rayyan.ai) for study selection and data management (Ouzzani et al. 2016). Two reviewers (L.P.B. and S.A.E.) independently screened titles and abstracts, retrieving full texts for studies meeting or potentially meeting inclusion criteria. Discrepancies were resolved by discussion or, when necessary, consultation with a third reviewer (M.H.A.S.). When data were missing or unclear, corresponding authors were contacted for clarification.

Data from each included study were extracted into standardized Excel sheets, capturing study design, sample size, patient demographics, recession characteristics, surgical technique, graft type, follow‐up duration, and main outcomes (mRC, %CRC, KTW gain, CAL gain, PD change, RES, and PROs, including patient‐reported postoperative morbidity). All entries were cross‐verified for accuracy and consistency.

### 2.5 Risk of Bias

The quality assessment of the included studies was carried out following the guidelines outlined in the Cochrane Handbook for Systematic Reviews of Interventions (version 6.1). RCTs were evaluated using the Cochrane RoB 2.0 tool (Sterne et al. 2019), which considers five domains of potential bias: (1) the randomization process, (2) deviations from intended interventions, (3) incomplete outcome data, (4) outcome measurement, and (5) selection of the reported results. Each domain was judged as presenting a low risk, some concerns, or a high risk of bias (RoB), leading to an overall risk rating for each study.

Two reviewers independently performed the risk of bias assessments. In cases of disagreement, consensus was reached through discussion, and a third reviewer was consulted when needed to resolve any conflicts.

### 2.6 Certainty (Quality) of Evidence

The certainty of evidence was assessed using the GRADE approach (Hultcrantz et al. 2017), starting from high certainty (all studies included were RCTs). Each outcome was then downgraded as appropriate for study limitations (risk of bias based on RoB 2), inconsistency (heterogeneity across trials), indirectness (applicability of populations, interventions, and outcomes), imprecision (95% CIs including no effect or small sample sizes), and other considerations such as publication bias. Final judgments were expressed as high, moderate, low, or very low certainty for each outcome.

### 2.7 Data Analysis

Data extraction and quantitative synthesis were performed at the study‐arm level for both experimental (DGG) and comparator (SCTG) groups, using the aggregate estimates reported by the original studies. Individual recession sites were not entered as independent observations in the meta‐analysis. For studies treating multiple recession defects within the same participant, the published aggregate estimates were used; therefore, adjustment for within‐patient correlation depended on the analytical methods applied in the original studies. No additional clustering adjustment was possible when the information required to estimate within‐patient correlation was not reported. Descriptive statistics summarized publication year, sample size, and surgical technique, as well as mean and standard deviation for each clinical outcome by graft type. For quantitative synthesis, a meta‐analysis dataset was constructed pairing DGG and SCTG arms from each study. For continuous outcomes—mean recession depth, root coverage percentage, keratinized tissue gain, clinical attachment level, probing depth, esthetic scores, and patient‐reported outcomes—mean differences (MDs) with 95% confidence intervals were calculated. Standard errors were derived from reported standard deviations and sample sizes. Random‐effects models using restricted maximum likelihood (REML) were applied, with Hartung–Knapp adjustment for small‐study correction and DerSimonian–Laird fallback when necessary. Between‐study heterogeneity was quantified with *I* ^2^, *τ* ^2^, and *Q* statistics. Subgroup analyses were planned by surgical technique (e.g., CAF vs. tunnel/envelope). Forest plots were generated for each outcome, and pooled results were tabulated. Unless otherwise specified, *p* values reported in the text refer to tests of the overall pooled treatment effect; *p* values displayed for heterogeneity in the forest plots correspond to Cochran's *Q* test. All analyses were conducted in RStudio (Version 2025.05.1 + 513) using the packages *meta*, *metafor*, *readr*, *dplyr*, *tidyr*, *stringr*.

## 3 Results

### 3.1 Article Selection

A total of 8372 records were identified across the five databases. After removal of 781 duplicates, 7591 records were screened, of which 7441 were excluded at the title and abstract stage. A total of 150 reports were sought for retrieval; all were retrieved and assessed for eligibility. Following full‐text assessment, 144 reports were excluded because of no direct comparison between DGG and SCTG (*n* = 133), abstract‐only publication (*n* = 3), wrong outcome (*n* = 3), wrong population (*n* = 2), clinical trial protocol (*n* = 1), duplicate publication (*n* = 1), or preliminary study for which the completed study was included (*n* = 1). A total of six studies were included in the qualitative and quantitative syntheses (Table 1).

**Table 1:** Included studies (Zucchelli et al. 2010; Bakhishov et al. 2021; Bertoldi et al. 2007; Cardoso et al. 2021; Mashaly et al. 2022; Zangrando et al. 2021) reporting general information and conclusions.

Study ID	Title	Authors	Year	Country	Study design	Sample size (*N*)	Total no. of recessions	Surgical technique	Follow‐up	Conclusions
1	De‐epithelialized gingival graft versus subepithelial connective tissue graft in the treatment of multiple adjacent gingival recessions using the tunnel technique: 1‐year results of a randomized clinical trial	Bakhishov H, Isler SC, Bozyel B, Yıldırım B, Tekindal MA, Ozdemir B	2021	Turkey	RCT	27	30	Tunnel	12	DGG + TUN resulted in higher mRC and CRC compared to SCTG + TUN; RES and PROMs outcome similar for both procedures
2	Late complications after root coverage with two types of subepithelial connective tissue grafts, clinical and histopathological evaluation: A prospective cohort study	Cardoso MV, Lara VS, Sant'Ana ACP, Damante CA, Ragghianti Zangrando MS	2020	Brazil	Prospective cohort study	60	133	CAF	12	5 complications in DGG and 1 in SCTG: white/yellowish discharge associated or not to gingival cul‐de‐sac
3	Soft tissue augmentation using de‐epithelialized free gingival graft compared to single‐line incision subepithelial connective tissue graft in the management of Miller Class I and II Gingival recession: A randomized controlled clinical trial	Mashaly M, Ghallab NA, Elbattawy W, Elarab AE	2022	Egypt	RCT	28	28	CAF	6	DFGG is a promising method for harvesting a CTG and significantly enhanced GT after 6 months
4	Clinical and patient‐centered outcomes using two types of subepithelial connective tissue grafts: A split‐mouth randomized clinical trial	Zangrando MSR, Eustachio RR, de Rezende MLR, Sant'ana ACP, Damante CA, Greghi SLA	2021	Brazil	RCT	21	84	CAF	6	DGG and SCTG presented satisfactory esthetic and clinical outcomes for multiple root coverage preocedures. SCTG has inferior morbidity compared to DGG
5	Patient morbidity and root coverage outcome after subepithelial connective tissue and de‐epithelialized grafts: A comparative randomized controlled clinical trial	Giovanni Zucchelli, Monica Mele, Martina Stefanini, Claudio Mazzotti, Matteo Marzadori, Lucio Montebugnoli, Massimo De Sanctis	2010	Italy	RCT	50	50	CAF	12	No differences were demonstrated in the post‐operative pain and root coverage outcome in patients subjected to CAF with CTG or DGG
6	Augmentation of keratinized gingiva through bilaminar connective tissue grafts: a comparison between two techniques	Bertoldi C, Bencivenni D, Lucchi A, Consolo U	2007	Italy	RCT	35	89	Nelson/Langer technique	12	This study did not show any statistical difference between the Nelson and the Langer technique as to root coverage and gain in keratinized gingiva

### 3.2 Qualitative Assessment

Included articles were published between 2007 and 2022, and all were RCTs. Cardoso et al. (2021) described randomization and allocation concealment with two parallel arm groups, though they reported their study as a “prospective cohort.” Both maxillary and mandibular recessions have been considered, with a total of 221 patients (mean age 36.09 ± 9.05; range 20–60). Smokers were excluded in all studies that reported smoking‐related eligibility criteria; Bertoldi et al. (2007) did not provide information regarding smoking status. The most frequent surgical technique performed to compare DGG versus SCTG is the CAF, reported in four studies (Zucchelli et al. 2010; Cardoso et al. 2021; Mashaly et al. 2022; Zangrando et al. 2021); one tunnel technique (Bakhishov et al. 2021), and one Nelson (modified by Harris) technique compared to the Langer surgical approach (Bertoldi et al. 2007) (Table 2).

**Table 2:** Demographic and recession‐defect data extracted.

Study_ID	Authors	Subjects/group	Male	Female	Age (mean)	(±SD)	Location (maxilla/mandible)	Recession type (single/multiple)	Surgical_technique	Graft	Smoking status
1	Bakhishov et al.	13	5	8	43.5	9.26	Maxilla/mandible	Multiple	Tunnel	DGG	Excluded
1	Bakhishov et al.	14	10	4	40.57	12.17	Maxilla/mandible	Multiple	Tunnel	SCTG	Excluded
2	Cardoso et al.	31	13	18	36.85	9.23	Maxilla/mandible	Multiple	CAF	DGG	Excluded
2	Cardoso et al.	29	12	17	37.52	8.41	Maxilla/mandible	Multiple	CAF	SCTG	Excluded
3	Mashaly et al.	14	11	17			Maxilla/mandible	Single/multiple	CAF	DGG	Excluded
3	Mashaly et al.	14					Maxilla/mandible	Single/multiple	CAF	SCTG	Excluded
4	Ragghianti‐Zangrado et al.	21	8	13	36.94		Maxilla/mandible	Single/multiple	CAF	DGG	Excluded
4	Ragghianti‐Zangrado et al.	21			36.94		Maxilla/mandible	Single/multiple	CAF	SCTG	Excluded
5	Zucchelli et al.	25	12	13	34.2	±6.8	Maxilla/mandible	Multiple	CAF	DGG	Excluded
5	Zucchelli et al.	25	10	15	32.2	±7.2	Maxilla/mandible	Multiple	CAF	SCTG	Excluded
6	Bertoldi et al.	19			26.1	6.2	Maxilla/mandible	Multiple	Nelson technique	SCTG	NA
6	Bertoldi et al.	15			26.1	6.2	Maxilla/mandible	Multiple	Langer technique	DGG	NA

### 3.3 Clinical and Patient‐Reported Outcomes

The pooled results for mRC, CRC, KTW gain, CAL change, PD change, RES, and patient‐reported pain/discomfort are summarized in Table 3.

**Table 3:** Summary of pooled meta‐analysis results comparing DGG with SCTG for treatment of Miller Class I/II gingival recession defects. Results are expressed as MD with 95% CI, estimated using REML. Negative values indicate outcomes favoring SCTG, while positive values indicate outcomes favoring DGG. Between‐study heterogeneity is presented as *I* ^2^.

Study_ID	Authors	Year	Sample_size	Subjects/group	Follow‐up_months	Surgical_technique	Graft	mRC	mRC_SD	CRC_%	CRC_%_SD	KT_gain	KT_gain_SD	CAL_change	CAL_change_SD	PD_change	PD_change_SD	RES	RES_SD	PROMs_7days	PROMs_7days_SD
1	Bakhishov et al.	2021	27	13	12	Tunnel	DGG	0.27	0.99	91.72	16.59	1.50	1.65	2.01	1.15	0.37	0.59	8.76	1.52	0.5	0.08
1	Bakhishov et al.	2021	27	14	12	Tunnel	SCTG	0.68	0.99	84.72	19.72	1.71	1.52	2.07	1.71	0.26	0.27	8.58	1.85	0.58	0.24
2	Cardoso et al.	2020	60	31	12	CAF	DGG	1.78	0.35	54.9		0.75	0.2	0.9	0.2	0.6	0.22				
2	Cardoso et al.	2020	60	29	12	CAF	SCTG	3.65	0.75	58.3		0.55	0.45	1.4	0.45	0.35	0.45				
3	Mashaly et al.	2022	28	14	6	CAF	DGG	0.07	0.27			3.64	0.84			0.5	0.24			3	2.65
3	Mashaly et al.	2022	28	14	6	CAF	SCTG	0.14	0.53			3.79	0.8			0.08	0.04			0.33	0.58
4	Ragghianti‐Zangrado et al.	2021	21	21	6	CAF	DGG	2.76	0.86	82.5	21.8	0.62	1.41	1.61	1.5	0.48	0.93	8.04	1.18	7.86	1.24
4	Ragghianti‐Zangrado et al.	2021	21	21	6	CAF	SCTG	2.62	0.97	78	22.2	0.84	1.03	1.98	1.29	0.09	1.07	8.36	1.17	4.66	2.31
5	Zucchelli et al.	2010	50	25	12	CAF	DGG	3.56	0.86	96.2	8.93	2.12	0.52	3.2	0.91	0.2	0.5			3.78	0.58
5	Zucchelli et al.	2010	50	25	12	CAF	SCTG	3.4	0.86	92.28	13.06	1.92	0.49	2.96	0.78	0.21	0.43			2.4	0.45
6	Bertoldi et al.	2007	35	19	12	Nelson technique	SCTG			93.86	11.8	4.18	1.07								
6	Bertoldi et al.	2007	35	15	12	Langer technique	DGG			91.99	13.8	4.57	0.95								

Meta‐analysis revealed no statistically significant difference in mRC between DGG and SCTG (*k* = 4; MD = −0.51 mm; 95% CI: −2.06 to 1.03; *p* = 0.368), with considerable between‐study heterogeneity (*I* ^2^ = 95.9%) (Figure 1a). In contrast, CRC significantly favored DGG (*k* = 3; MD = 4.45%; 95% CI: 1.21–7.69; *p* = 0.027; *I* ^2^ = 0%) (Figure 1b). KTW gain showed a small numerical difference favoring DGG (*k* = 5; MD = 0.16 mm; 95% CI: 0.00–0.32; *p* = 0.053; *I* ^2^ = 0%), although the effect did not reach statistical significance (Figure 1c).

Figure 1(a) Forest plot of mRC comparing DGG versus SCTG. Effect sizes are expressed as MD in millimeters with 95% CI. Negative values favor SCTG, positive values favor DGG. Pooled estimates were obtained using REML. (b) Forest plot of CRC% comparing DGG and SCTG. Effect sizes are expressed as mean differences in percent coverage with 95% CI. Positive values indicate higher coverage with DGG. (c) Forest plot of KTW gain comparing DGG and SCTG. Effect sizes are expressed as mean differences in millimeters with 95% CI. Positive values favor DGG. (d) Forest plot of CAL change comparing DGG and SCTG. Effect sizes are expressed as mean differences in millimeters with 95% CI. Negative values favor SCTG. (e) Forest plot of PD change comparing DGG and SCTG. Effect sizes are expressed as mean differences in millimeters with 95% CI. Positive values favor DGG. (f) Forest plot of RES comparing DGG and SCTG (if reported). Effect sizes are expressed as mean differences with 95% CI. Higher values indicate better esthetic outcomes. (g) Forest plot of PROs at 7 days comparing DGG and SCTG. Effect sizes are expressed as mean differences in reported pain/discomfort scores with 95% CI. Negative values favor DGG (lower morbidity).

**Figure:**
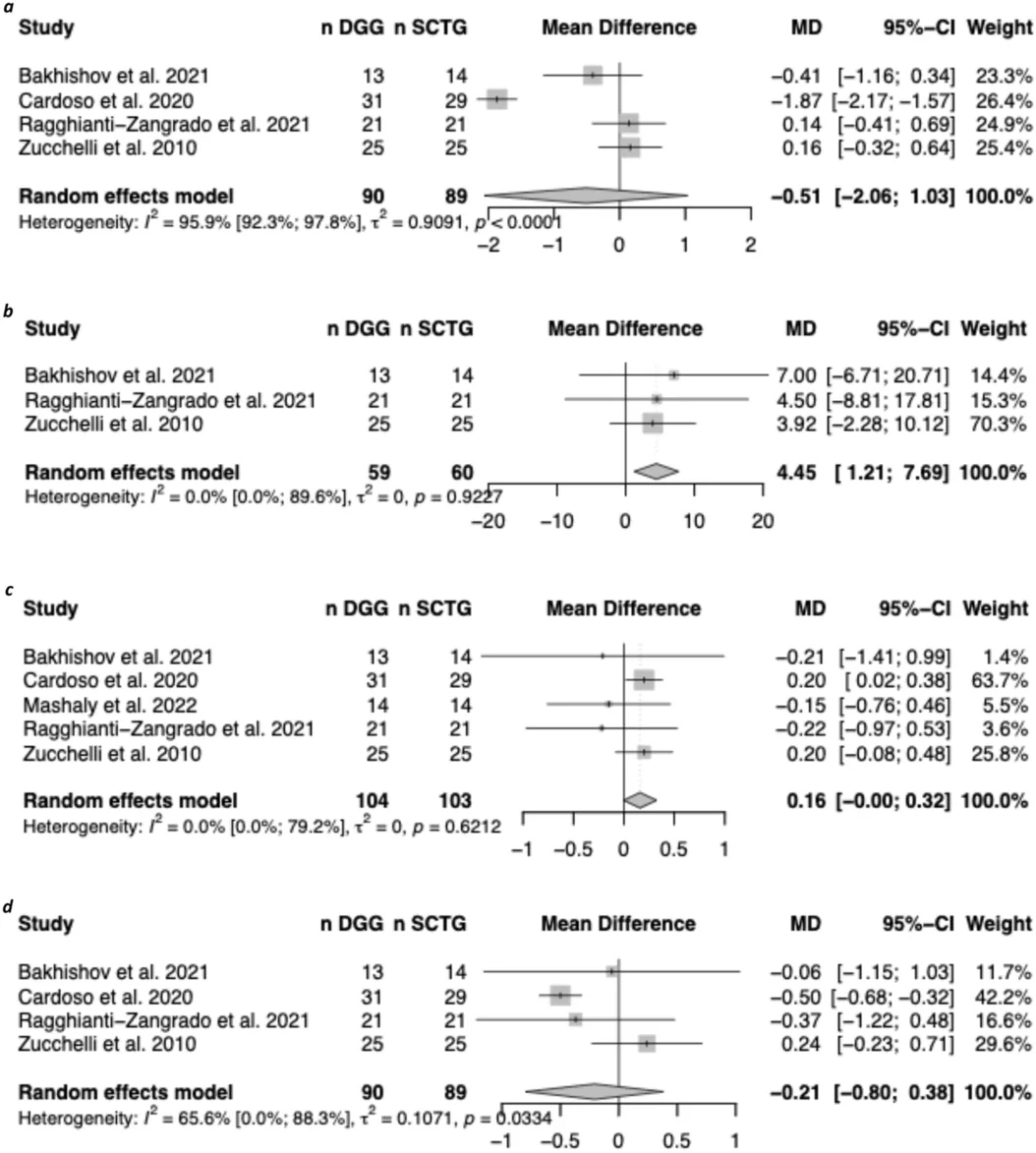

**Figure:**
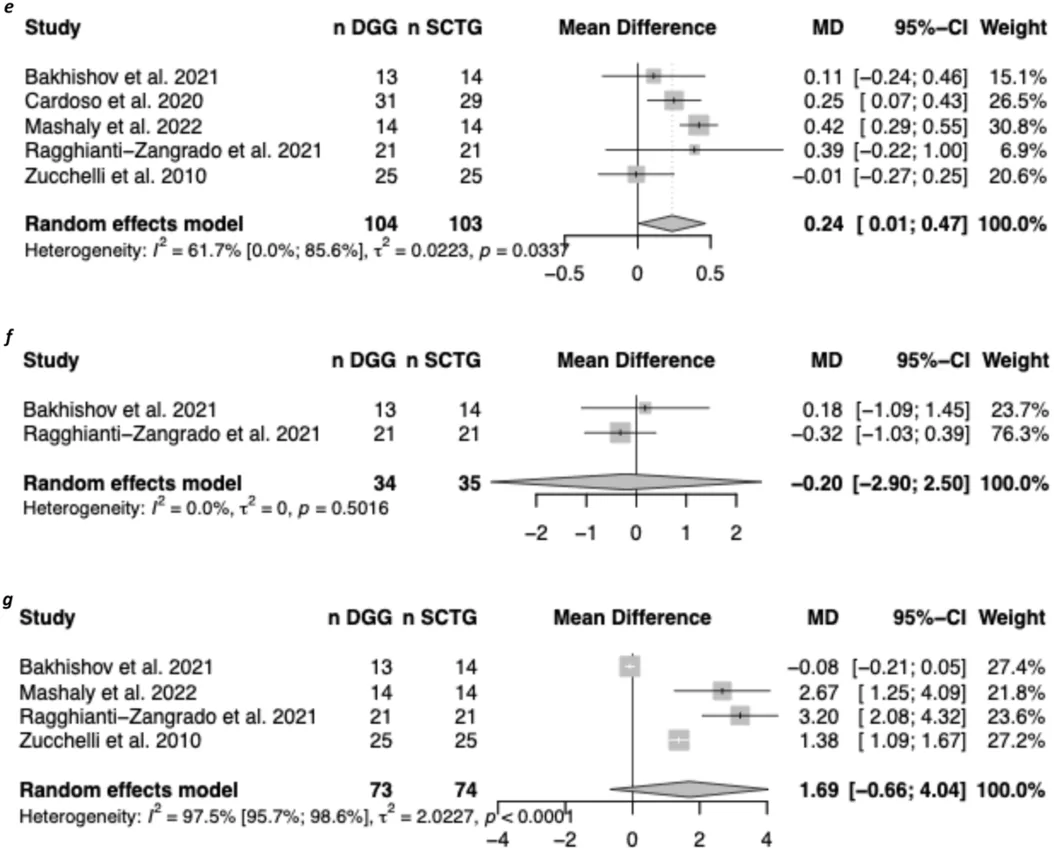

CAL change did not differ significantly between groups (*k* = 4; MD = −0.21 mm; 95% CI: −0.80 to 0.38; *p* = 0.341), with substantial heterogeneity (*I* ^2^ = 65.6%) (Figure 1d). PD change showed a small statistically significant difference favoring DGG (*k* = 5; MD = 0.24 mm; 95% CI: 0.01–0.47; *p* = 0.045), although substantial heterogeneity was present (*I* ^2^ = 61.7%) (Figure 1e). RES showed no statistically significant difference between grafting approaches (*k* = 2; MD = −0.20; 95% CI: −2.90 to 2.50; *p* = 0.518; *I* ^2^ = 0%) (Figure 1f); however, this estimate was based on only two studies and was imprecise.

Patient‐reported pain/discomfort (PROs) at 7 days was available from four studies. No statistically significant difference was observed between DGG and SCTG (*k* = 4; MD = 1.69; 95% CI: −0.66 to 4.04; *p* = 0.106), with considerable between‐study heterogeneity (*I* ^2^ = 97.5%) (Figure 1g). Accordingly, the pooled estimates, particularly those characterized by substantial heterogeneity or based on few studies, should be interpreted cautiously.

Substantial between‐study heterogeneity was observed for mRC, CAL change, and PD change. Although surgical technique was considered as a potential source of this variability, formal subgroup analysis was not undertaken because four studies employed CAF, whereas the tunnel and Nelson/Langer approaches were represented by only one study each, preventing reliable technique‐specific pooled comparisons.

### 3.4 Risk of Bias

The risk of bias (RoB 2) assessment across the six included RCTs revealed a mixed quality of evidence (Figure 2). Two trials were judged at low risk of bias, while several studies presented some concerns, mainly related to the randomization process, selective reporting, or measurement of outcomes. Across studies, the randomization process and outcome measurements were the most frequent sources of potential bias, whereas deviations from intended interventions and missing outcome data were generally low.

**Figure 2:**
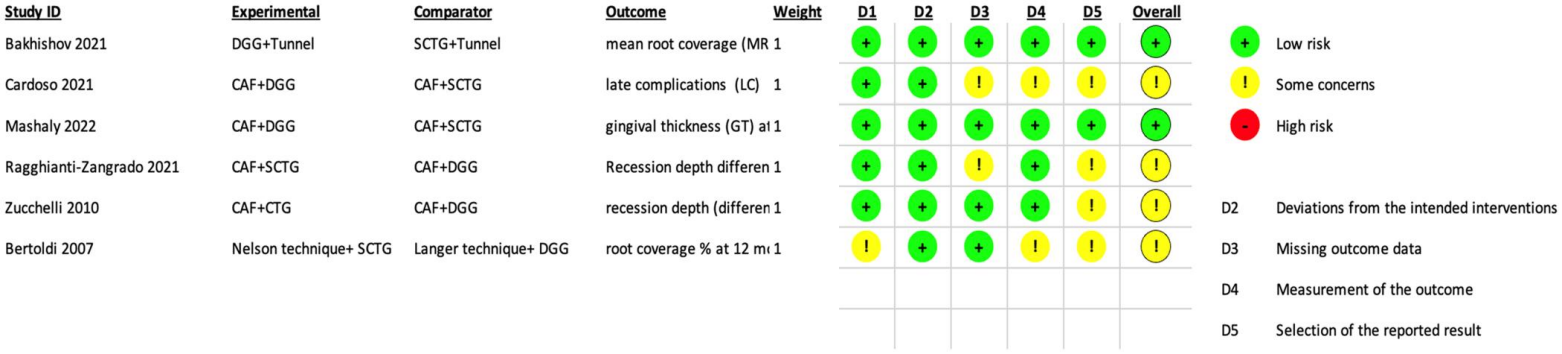
Traffic light plot of the risk‐of‐bias assessment using the Cochrane RoB 2 tool.

### 3.5 Certainty of Evidence

The GRADE assessment indicated that the certainty of evidence comparing DGG with SCTG was overall limited, with most outcomes rated as low certainty (Table 4). Moderate‐certainty evidence suggested that DGG may increase CRC by approximately 4.45% compared with SCTG (95% CI: 1.21–7.69). The certainty of evidence for mRC, KTW gain, CAL change, PD change, and RES was rated as low, primarily because of concerns regarding risk of bias and imprecision. Although the pooled estimate for PD change did not cross the line of no effect, the magnitude of the effect was small (MD = 0.24 mm; 95% CI: 0.01–0.47), and the certainty of evidence remained low. For patient‐reported pain/discomfort at 7 days, the certainty of evidence was very low because of concerns regarding risk of bias, serious inconsistency, and imprecision, with substantial between‐study heterogeneity (*I* ^2^ = 97.5%).

**Table 4:** This summary of findings (SoF) table presents the GRADE evaluation of evidence comparing DGG versus SCTG for the treatment of gingival recessions.

Certainty assessment							No. of patients		Effect		Certainty	Importance
No. of studies	Study design	Risk of bias	Inconsistency	Indirectness	Imprecision	Other considerations	DGG	SCTG	Relative (95% CI)	Absolute (95% CI)		
Complete root coverage (CRC%)												
3	Randomized trials	Serious	Not serious	Not serious	Not serious	None	105	108	—	MD 4.45 (95% CI 1.21–7.69)	⨁⨁⨁◯ Moderate	IMPORTANT
Mean root coverage (mRC)												
4	Randomized trials	Serious	Not serious	Not serious	Serious	None	104	103	—	MD −0.51 (95% CI −2.06 to 1.03)	⨁⨁◯◯ Low	IMPORTANT
Keratinized tissue width (KTW) gain												
5	Randomized trials	Serious	Not serious	Not serious	Serious	None	119	122	—	MD 0.16 (95% CI −0.00 to 0.32)	⨁⨁◯◯ Low	IMPORTANT
Clinical attachment level (CAL) change												
4	Randomized trials	Serious	Not serious	Not serious	Serious	None	90	89	—	MD −0.21 (95% CI −0.80 to 0.38)	⨁⨁◯◯ Low	IMPORTANT
Probing depth (PD) change												
5	Randomized trials	Serious	Not serious	Not serious	Serious	None	104	103	—	MD 0.24 (95% CI 0.01–0.47)	⨁⨁◯◯ Low	IMPORTANT
Esthetic outcome (RES)												
2	Randomized trials	Serious	Not serious	Not serious	Serious	None	48	48	—	MD −0.20 (95% CI −2.90 to 2.50)	⨁⨁◯◯ Low	IMPORTANT
PROs at 7 days (pain/morbidity)												
4	Randomized trials	Serious	Serious	Not serious	Serious	None	73	74	—	MD 1.69 (95% CI −0.66 to 4.04)	⨁◯◯◯ Very low	IMPORTANT

## 4 Discussion

This systematic review and meta‐analysis included six studies directly comparing DGG with SCTG for the treatment of RT1 gingival recessions. Overall, the available evidence suggests relatively small differences between the two graft‐harvesting approaches. No statistically significant differences were observed for mRC, KTW gain, CAL change, RES, or patient‐reported pain/discomfort, whereas DGG showed a modest advantage for CRC and a small difference in PD change. However, these findings should be interpreted cautiously given the limited number of studies, substantial heterogeneity for several outcomes, and the predominantly low certainty of evidence according to GRADE. Thus, the current evidence does not support broad clinical superiority of either harvesting approach.

The present findings should be interpreted in the context of the previous systematic review and meta‐analysis by Tavelli et al. (2019). As acknowledged by the authors (Tavelli et al. 2019), because of the limited number of RCTs directly comparing SCTG and DGG, studies evaluating the two grafting approaches were pooled separately for each outcome. Thus, their quantitative synthesis primarily compared pooled estimates derived from CAF + SCTG and CAF + DGG studies rather than being restricted to head‐to‐head randomized comparisons.

Despite these methodological differences, the present findings are broadly consistent with those of Tavelli et al. (2019) suggesting relatively small differences between DGG and SCTG across several clinical outcomes. The present review extends the previous evidence by incorporating subsequently published direct comparative studies, applying the contemporary RT1 framework, considering different bilaminar root‐coverage approaches, and including clinician‐ and patient‐centered outcomes together with an assessment of certainty using GRADE. Within this direct comparative framework, the modest advantage observed for CRC should be interpreted as a refinement of the existing evidence rather than evidence of overall superiority of DGG.

The clinical interest in different CTG‐harvesting approaches reflects the need to preserve the predictable outcomes associated with autogenous connective tissue while minimizing donor‐site morbidity. Although several soft‐tissue substitutes have been proposed to eliminate the need for a second surgical site, autogenous CTG remains an important reference approach because alternative materials have not consistently demonstrated comparable clinical outcomes (de Queiroz Côrtes et al. 2006; Stefanini et al. 2016). Unfortunately, these alternative grafting materials have not demonstrated better or comparable outcomes to autologous CTGs (Cairo et al. 2014, 2016; Chambrone and Tatakis 2015). Thus, there is a constant search in the periodontal literature for efficient CTG‐harvesting techniques to minimize patients' morbidity without compromising root coverage outcomes. This led to the emergence of the DGG by Zucchelli et al. (2010), through which we can obtain denser, firmer, and more stable graft tissue than the standard SCTG (Bertl et al. 2015). Many authors preferred the use of SCTG as opposed to DGG merely due to its ability to heal by primary intention, which has been shown to decrease morbidity as contrasted with open wound healing (Parma‐Benfenati et al. 2025; Del Pizzo et al. 2002; Wessel and Tatakis 2008). However, Zucchelli et al. proved that when uniform grafts are harvested, and the palate is properly managed and protected, both DGG and SCTG‐harvesting techniques demonstrated similar PROs (Zucchelli et al. 2010). Differences in donor‐site healing between DGG and SCTG could theoretically influence postoperative morbidity; however, the present pooled analysis did not identify a statistically significant difference in early patient‐reported pain/discomfort, and the substantial heterogeneity of this outcome precludes firm conclusions.

Once the harvesting approach was demonstrated to be a non‐influencing factor of patient morbidity, the question then becomes whether the clinical outcomes of periodontal plastic surgery are influenced by a DGG or SCTG approach (Zucchelli et al. 2010). It is assumed that DGG would risk including some of the epithelium in the graft. A classic histologic study in humans reported that epithelial remnants were found in 80% of SCTGs, yet it did not affect the root coverage outcomes (Harris 2003). A more recent study demonstrated that roughly 80% of the DGG samples were actually epithelium‐free, whether de‐epithelialized intraorally or extra‐orally (Couso‐Queiruga et al. 2023).

A successful mucogingival therapy relies primarily on complete resolution of the defect (Wennström 1996; de Sanctis and Zucchelli 2007). Nevertheless, CRC, minimal probing depths, KTW gain, and esthetic outcomes are the main factors of successful mucogingival therapy (Tonetti et al. 2025). The results of this review demonstrated that both grafts are effective for root coverage procedures in RT1 defects. While mRD and CAL outcomes were equivalent, DGG showed a slight advantage in %CRC (4.45% [95% CI: 1.21–7.69; *p* = 0.027; *I* ^2^ = 0%]) and =0.25 mm PD reduction. Keratinized tissue gain was comparable, though a small numerical advantage favored DGG.

The RES score encompasses assessment of the gingival margin level, marginal tissue contour, soft tissue texture, mucogingival alignment, and gingival color (Cairo et al. 2009). Our results showed that RES were similar between groups. Also, patient satisfaction was proposed as an additional factor for success (Stefanini et al. 2016; Roman et al. 2012). From our analysis of PROs at 7 days, DGG was associated with higher morbidity than SCTG, although the overall effect was not statistically significant.

An additional consideration when selecting a graft harvesting technique is the potential for graft‐related soft tissue complications. De‐epithelialized CTGs have been associated with a higher incidence of epithelial overgrowth phenomena, including reepithelialization, epithelial bands, cul‐de‐sac formation, and keloid‐like tissue proliferation (Ripoll et al. 2021). According to the study by Zucchelli et al. (2014), keloid formation due to graft exposure was responsible for the worse color match evaluation when comparing CAF + CTG or CAF alone, with a percentage up to 44% in a 5‐year follow‐up evaluation. Therefore, clinicians should carefully weigh the potential benefits of DGG and its meticulous de‐epithelialization stage. Further long‐term, well‐designed clinical trials with standardized reporting of soft tissue complications are needed to better clarify the clinical relevance of these findings.

Although our methodology was quite different, our results are not much different from those of the review by Tavelli et al. (2019). While their review demonstrated higher mRC for the DGG than SCTG, both reviews concluded that DGG significantly favored %CRC, which also agreed with Zucchelli et al. who showed a CRC of 96% for DGG and 92% for the SCTG group (Zucchelli et al. 2010). Both reviews also agreed that DGG provided a borderline advantage for KTW gain.

The difference in the KTW gains between the two techniques is probably due to the different nature of the grafts. DGG is mainly composed of lamina propria and, thus, characterized by a greater amount of fibrous connective tissue (Bertl et al. 2015). These properties make DGG firmer, more stable, and easier to manage than SCTG (Zucchelli and Mounssif 2015). Also, the connective tissue composition can influence the differentiation of the overlying epithelial layer (Karring et al. 1975), and deeper palatal connective tissue may be less capable of inducing keratinization of the epithelium (Ouhayoun et al. 1988). In 1968, Sullivan and Atkins suggested that fatty tissue should be completely removed from the graft, due to its inclination to act as a barrier to diffusion and vascularization (Sullivan and Atkins 1968). Also, in 1985, Miller speculated that the submucosa could prevent a direct interface between connective tissue and the root (Miller 1985b).

Based on this previous assumption, two studies investigated the effect of the CTG orientation on gingival augmentation without observing any significant differences (Al‐Zahrani et al. 2004; Lafzi et al. 2007). The absence of difference could, again, be explained by the fact that graft composition, rather than orientation. The graft composition may be what in fact play a role in stimulating the differentiation of the epithelial layer. These findings agree with Cordioli et al. who reported a lack of transformation of the mucosa into KTW when bilaminar techniques involved SCTG (Cordioli et al. 2001). Moreover, a recent systematic review from Sculean et al. (2014) concluded that CTG from the deep palate does not have the same potential of inducing keratinization compared to a more superficial CTG. Furthermore, the composition of the CTG is also responsible for graft shrinkage, which, in turn, can alter the amount of KTW found, resulting in less post‐operative shrinkage with DGG compared to SCTG (Zucchelli et al. 2010).

In our study, CAL changes were not significantly different between groups. However, PD changed slightly in favor of the DGG group. This could be attributed to the more fibrous nature of the CT obtained with the DGG, which could enhance the attachment between the DGG and the tooth surface, as suggested by Miller (1985b). All the studies included in our systematic review confirmed this.

Due to the lack of available data, late complications were not included in our analysis. However, Ripoll et al. (2021) conducted an RCT reporting 1‐year postoperative complications for both techniques. Complications were categorized as major (reepithelialization, cul‐de‐sac formation, epithelial bands, cysts, and exostoses) or minor (revascularization, discoloration). No major complications were observed in the SCTG group, whereas in the DGG group, reepithelialization, cul‐de‐sac formation, and epithelial bands occurred in 15.6% of cases each. Minor complications were more frequent with DGG, showing 31.3% revascularization and 46.9% discoloration compared to 5.9% and 14.7%, respectively, in the SCTG group (Ripoll et al. 2021).

Several limitations should be considered when interpreting the present findings. First, only six studies met the eligibility criteria, and individual meta‐analyses were based on as few as two to five studies, limiting the precision and robustness of the pooled estimates and precluding reliable assessment of publication bias. Second, substantial between‐study heterogeneity was observed for several outcomes, particularly mRC, CAL change, PD change, and patient‐reported pain/discomfort. Differences in surgical technique, treatment of single versus multiple recessions, anatomical characteristics, graft harvesting and management, follow‐up duration, and outcome assessment may have contributed to this variability; however, the limited number of studies precluded reliable exploration through subgroup analysis or meta‐regression. Third, all included investigations were single‐center, university‐based studies, which may limit generalizability to routine clinical settings. Moreover, two of the six studies originated from the Bauru School of Dentistry, University of São Paulo (Cardoso et al. 2021; Zangrando et al. 2021) and involved overlapping investigators; although they used distinct study designs and sample sizes, patient‐level overlap could not be unequivocally excluded, and shared institutional practices may have reduced the effective diversity of the evidence base. Fourth, the certainty of evidence was predominantly low according to GRADE and very low for patient‐reported pain/discomfort, limiting confidence in several pooled estimates. Finally, long‐term data on graft‐related complications were sparse and could not be quantitatively synthesized. These limitations warrant cautious interpretation of the findings and restrict their generalizability beyond the populations and clinical settings represented in the available studies.

Future research should prioritize adequately powered, multicenter randomized trials directly comparing DGG and SCTG across diverse clinical settings and operators. Standardization of surgical protocols, outcome definitions, and reporting would facilitate more reliable comparisons, while longer follow‐up is needed to characterize the incidence and clinical relevance of graft‐related complications. Future studies should also consistently evaluate patient‐reported pain/discomfort and esthetic outcomes and report sufficient methodological information to account for clustering when multiple recession defects are treated within the same patient.

In summary, current direct comparative evidence suggests that DGG and SCTG provide broadly similar clinical, esthetic, and patient‐reported outcomes for the treatment of RT1 gingival recessions, with a modest advantage for DGG in CRC and a small difference in PD change. Given the limited evidence base, heterogeneity across studies, and generally low certainty of evidence, these differences should not be interpreted as demonstrating overall superiority of one harvesting technique. Clinical selection should therefore remain individualized until more robust multicenter and long‐term comparative evidence becomes available.

## 5 Conclusions

This systematic review suggests that DGG and SCTG are both effective treatment options for RT1 gingival recessions, with no statistically significant differences observed for most evaluated clinical, esthetic, and patient‐reported outcomes. DGG showed a modest advantage in complete root coverage and a small statistically significant difference in probing depth change; however, the clinical relevance of these differences remains uncertain. These findings should be interpreted cautiously given the small number of included studies, the limited number of studies contributing to individual meta‐analyses, substantial heterogeneity for several outcomes, and the predominantly low certainty of evidence. Further adequately powered, multicenter randomized controlled trials with standardized outcome reporting and long‐term follow‐up are needed to confirm these findings and better define the relative clinical advantages of the two graft‐harvesting approaches.

## Author Contributions

**Lucrezia Parma Benfenati:** project management, supervision, data curation, investigation, formal analysis, statistical analysis, writing – original draft preparation, writing – review and editing. **Saeed Abdelaziz ElRefaei:** data curation, investigation, writing – original draft preparation, writing – review and editing. **Muhammad H. A. Saleh:** conceptualization, supervision, writing – review and editing. **Hom‐Lay Wang:** writing – review and editing.

## Funding

The authors have nothing to report.

## Conflicts of Interest

The authors declare no conflicts of interest.

## Supporting information


Supporting File

## Data Availability

The data supporting this study's findings are available from the corresponding author, L.P.B., upon reasonable request.
