# Supplementary material for: Efficacy of De‐Epithelialized vs. Subepithelial Grafts in Periodontal Plastic Surgery for Gingival Recession: A Systematic Review and Meta‐Analysis of Root Coverage Techniques
Source: Clin Exp Dent Res. 2026 Nov 9;12(6):e70499. doi: 10.1002/cre2.70499 (PMC13747200; doi:10.1002/cre2.70499)
Supplement: Supplementary file 1 [file CRE2-12-e70499-s001.docx]

# 1. MEDLINE (PubMed) Search Strategy

("Gingival Recession"[MeSH] OR "Recession"[All Fields] OR "Gingival Recession"[All Fields])
AND
("Periodontal Plastic Surgery"[MeSH] OR "Mucogingival Surgery"[All Fields] OR "Root Coverage"[All Fields] OR "Coronally Advanced Flap"[All Fields] OR "Tunnel Technique"[All Fields] OR "Envelope Technique"[All Fields])
AND
(("Connective Tissue Graft"[MeSH] OR "Connective Tissue Graft"[All Fields] OR "SCTG"[All Fields])
OR
("De-epithelialized Gingival Graft"[All Fields] OR "DGG"[All Fields] OR "De-epithelialized Graft"[All Fields]))
AND
("Randomized Controlled Trial"[Publication Type] OR "Randomized Controlled Trial"[All Fields] OR "RCT"[All Fields])
AND
("Humans"[MeSH] AND English[lang])

Optional date filter for recent studies:

AND ("2018/01/01"[Date - Publication] : "3000"[Date - Publication])

(((((((((((((((((((((((gingival recession[MeSH Terms]) OR (gingival recession)) OR (recession)) OR (mucogingival defect)) AND (plastic surgery[MeSH Terms])) OR (periodontal plastic surgery)) OR (mucogingival surgery)) OR (periodontal surgery)) OR (root coverage)) OR (coronally advanced flap)) OR (CAF)) OR (Tunnel technique)) OR (envelope flap)) OR (envelope technique)) AND (connective tissue[MeSH Terms])) OR (connective tissue graft)) OR (SCGT)) OR (sub-epithelial connective tissue graft)) OR (de-epithelialized gingival graft)) OR (DGG)) OR (de-epithelialized graft)) OR (de-epithelialized free-gingival graft)) OR (de-epithelialized FGG)) 54,838

Clinical Study; Clinical Trial; Clinical Trial Protocol; Comparative Study; Controlled Clinical Trial; Randomized Controlled Trial 7,194

Humans 5,628

# 2. EMBASE Search Strategy

('gingival recession'/exp OR 'gingival recession':ti,ab)
AND
('periodontal plastic surgery'/exp OR 'mucogingival surgery':ti,ab OR 'root coverage':ti,ab OR 'coronally advanced flap':ti,ab OR 'tunnel technique':ti,ab OR 'envelope technique':ti,ab)
AND
(('connective tissue graft'/exp OR 'connective tissue graft':ti,ab OR 'SCTG':ti,ab)
OR
('de-epithelialized gingival graft':ti,ab OR 'DGG':ti,ab OR 'de-epithelialized graft':ti,ab))
AND
('randomized controlled trial'/exp OR 'randomized controlled trial':ti,ab OR 'RCT':ti,ab)
AND
[humans]/lim AND [english]/lim

Optional date filter:

AND [2018-2025]/py

319 results

# 3. Cochrane CENTRAL Search Strategy

("gingival recession"):ti,ab,kw
AND
("root coverage" OR "periodontal plastic surgery" OR "mucogingival surgery" OR "coronally advanced flap" OR "tunnel technique" OR "envelope technique"):ti,ab,kw
AND
(("connective tissue graft" OR SCTG):ti,ab,kw
OR
("de-epithelialized gingival graft" OR DGG OR "de-epithelialized graft"):ti,ab,kw)

Note: CENTRAL contains only trials; no RCT filter is needed. Use built-in filters to restrict by publication date if desired.

2424 results
